# Valacyclovir-associated neurotoxicity among patients on hemodialysis and peritoneal dialysis: A nationwide population-based study

**DOI:** 10.3389/fmed.2022.997379

**Published:** 2022-09-20

**Authors:** Yi-Chun Wang, Shu-Hui Juan, Ching-Hao Li, Chu-Lin Chou, Li-Ying Chen, Li-Nien Chien, Te-Chao Fang

**Affiliations:** ^1^Division of Nephrology, Taipei Tzu Chi Hospital, Buddhist Tzu Chi Medical Foundation, New Taipei City, Taiwan; ^2^School of Medicine, Tzu Chi University, Hualien, Taiwan; ^3^Department of Physiology, School of Medicine, College of Medicine, Taipei Medical University, Taipei, Taiwan; ^4^TMU Research Center of Urology and Kidney, Taipei Medical University, Taipei, Taiwan; ^5^Division of Nephrology, Department of Internal Medicine, School of Medicine, College of Medicine, Taipei Medical University, Taipei, Taiwan; ^6^Division of Nephrology, Department of Internal Medicine, Hsin Kuo Min Hospital, Taipei Medical University, Taoyuan City, Taiwan; ^7^Division of Nephrology, Department of Internal Medicine, Shuang Ho Hospital, Taipei Medical University, New Taipei City, Taiwan; ^8^Health Data Analytics and Statistics Center, Office of Data Science, Taipei Medical University, Taipei, Taiwan; ^9^Institute of Health and Welfare Policy, College of Medicince, National Yang Ming Chiao Tung University, Taipei, Taiwan; ^10^Graduate Institute of Data Science, College of Management, Taipei Medical University, Taipei, Taiwan; ^11^Division of Nephrology, Department of Internal Medicine, Taipei Medical University Hospital, Taipei Medical University, Taipei, Taiwan

**Keywords:** valacyclovir, neurotoxicity, hemodialysis (HD), peritoneal dialysis, population-based study

## Abstract

Whether valacyclovir-associated neurotoxicity (VAN) occurs more frequently in patients with end-stage renal disease (ESRD) on dialysis is unknown. This is the first population-based study to examine the risk of VAN associated with ESRD patients on dialysis. Among 2,284,800 patients diagnosed as having herpes zoster from 2002 to 2016, patients with ESRD on dialysis and individuals with normal renal function were enrolled in this study. Following propensity score matching, we compared the risk of altered mental status between valacyclovir users and non-users in the ESRD and normal renal function cohorts over a 30-day follow-up period. In the ESRD cohort, the incidence of altered mental status was 1.68 and 0.52 per 1,000 person-day in valacyclovir users and non-users, respectively, with an adjusted hazard ratio (HR) of 3.22 (95% confidence interval [CI]: 2.04–4.99, *P* < 0.001). The incidence of altered mental status of valacyclovir users on hemodialysis (HD) and peritoneal dialysis (PD) was higher than that of non-users. The adjusted HR was 3.20 (95% CI: 1.98–5.15, *P* < 0.001) for those on HD and 3.44 (95% CI: 1.13–10.49, *P* = 0.030) for those with PD. However, altered mental status was not observed in patients on HD receiving ≤500 mg of valacyclovir three times per week or in those on PD receiving ≤500 mg of valacyclovir per day. The findings demonstrate that adjusting the valacyclovir dosage and monitoring VAN in patients with HD and PD who have herpes zoster is crucial.

## Introduction

Varicella-zoster virus (VZV) causes a primary infection known as chickenpox or varicella and remains dormant in peripheral neurons. VZV is reactivated in the host at an older age or when their body is in an immunosuppressed state. VZV reactivation causes herpes zoster, which is characterized by painful and cutaneous eruptions with a dermatomal distribution. One study indicated that patients with renal failure who may be immunosuppressed had a higher risk of herpes zoster than did the general population (1).

Herpes zoster is usually treated with antiviral therapy and analgesics. Indications of antiviral treatment include older age, severe pain or rash, face or eye involvement, other complications of herpes zoster, or a weakened immune system (2). Antiviral agents, including acyclovir, valacyclovir, and famciclovir are used in the treatment of acute herpes zoster infection. Valacyclovir is preferred in clinical practice because it entails less frequent dosing compared with acyclovir and lower costs compared with famciclovir (2). In one study, valacyclovir was confirmed to alleviate the pain related to herpes zoster; moreover, it had a similar safety profile to that of acyclovir (3).

Because valacyclovir is excreted through the kidneys, patients with impaired renal function might be the most susceptible to valacyclovir-associated neurotoxicity (VAN). Several case reports have documented the occurrence of VAN in patients with end-stage renal disease (ESRD) who are on dialysis, including hemodialysis (HD) and peritoneal dialysis (PD) (4–11). To the best of our knowledge, the risk of VAN in a population-based study, including the general population and patients on dialysis, has rarely been investigated. Therefore, we estimated herein the incidence of significant neurotoxicity (altered mental status) after valacyclovir use among patients with ESRD on HD and PD and among individuals with normal renal function. Moreover, we examined the association between the risk of VAN and valacyclovir use in both cohorts.

## Materials and methods

### Data source

Data were retrieved from the National Health Insurance (NHI) Research Database (NHIRD), Taiwan’s population-based medical claims database. Established in 1995, the NHI program covers more than 99% of the Taiwanese population. The NHIRD contains medical information such as inpatient and outpatient visits, disease diagnosis, and medication prescriptions. One of the largest medical databases worldwide, it has been used in numerous observational studies (12–19). Data from 2000 to 2016 were analyzed in this study. In the NHIRD, diagnoses before 2015 are based on the *International Classification of Diseases, Ninth Revision, Clinical Modification* (*ICD-9-CM*), and those made after 2016 are based on the *International Classification of Diseases, Tenth Revision, Clinical Modification* (*ICD-10-CM*). Data on prescribed medications are classified according to the Anatomical Therapeutic Chemical classification system. The accuracy of the diagnoses and treatment rationale is routinely reviewed by the NHI Administration. All personally identifiable information was encrypted before the data were released. Therefore, the Institutional Review Board (IRB) of the Taipei Tzu Chi Hospital (IRB number: 03-W02-091) waived the need for informed consent.

### Study design and cohort

**FIGURE 1:**
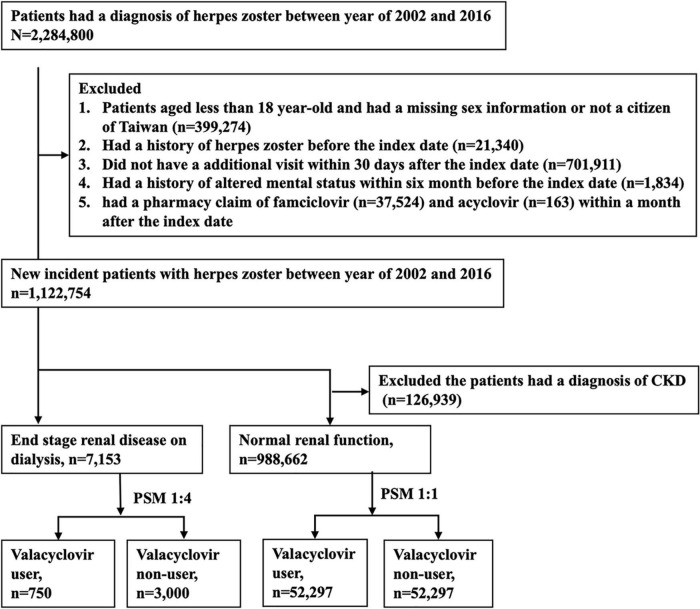
Patient selection process. PSM, propensity score matching.

This study adopted a population-based retrospective design. Figure 1 displays the participant selection process. Patients with a diagnostic claim for herpes zoster (*ICD-9-CM*: 053.x or *ICD-10-CM*: B02) between 2002 and 2016 were first included, and the date of the first diagnosis was defined as the index date. We excluded patients who (1) were aged younger than 18 years, (2) had missing sex information or were not Taiwanese citizens, (3) had a history of herpes zoster before the index date, (4) did not make follow-up medical visits in the 30-day period following the index date, or (5) had a history of altered mental status in the 6-month period before the index date. In addition, patients prescribed famciclovir or acyclovir in the 30-day period following the index date were excluded. According to NHI reimbursement regulations, indications of valacyclovir for herpes zoster in Taiwan include face, eye, or genital involvement and an immunocompromised status.

Patients who had continuously received dialysis for more than 3 months before the index date were classified into the ESRD cohort. Individuals with no diagnosis of chronic kidney disease (CKD) before the index date were classified into the normal renal function cohort. We adopted the propensity score matching (PSM) approach to select two groups of patients with similar baseline characteristics but different valacyclovir use status in each cohort. PSM is commonly applied in epidemiologic observational studies to minimize sample selection bias (20–22). Valacyclovir users and non-users were matched at a ratio of 1:4 in the ESRD cohort and at a ratio of 1:1 in the normal renal function cohort. A propensity score was estimated on the basis of variables associated with altered mental status, including age; sex; and a history of diabetic mellitus, coronary artery disease, congestive heart failure, peripheral vascular disease, cerebrovascular disease, liver cirrhosis, or cancer (Table 1). We also used the Charlson comorbidity index (CCI) as a proxy to adjust for the severity of previous or coexisting conditions. The diagnostic codes are listed in Supplementary Table 1.

**TABLE 1:** Baseline characteristics of patients with herpes zoster receiving or not receiving valacyclovir after propensity score matching.

	End-stage renal disease (on dialysis; PSM 1:4)					Normal renal function (PSM 1:1)				
	User		Non-user		SMD	User		Non-user		SMD
	*n*	(%)	*n*	(%)		*n*	(%)		(%)	
Sample size	750	(100.0)	3,000	(100.0)		52,297	(100.0)	52,297	(100.0)	
**Type of dialysis before diagnosis**										
HD	637	(84.9)	2,558	(85.3)	0.01					
PD	113	(15.1)	442	(14.7)	0.01					
Age, mean ± SD (years)	65.1 ± 12.2		65.1 ± 12.1		<0.01	57.2 ± 16.9		56.7 ± 16.8		0.03
18–44	39	(5.2)	147	(4.9)	0.01	11,908	(22.8)	11,909	(22.8)	<0.01
45–64	313	(41.7)	1,242	(41.4)	0.01	22,163	(42.4)	22,164	(42.4)	<0.01
65+	398	(53.1)	1,611	(53.7)	0.01	18,226	(34.9)	18,224	(34.8)	<0.01
Male sex	317	(42.3)	1,234	(41.1)	0.02	25,257	(48.3)	25,259	(48.3)	<0.01
**Previous comorbidity**										
Diabetes mellitus	267	(35.6)	1,030	(34.3)	0.03	7,273	(13.9)	7,273	(13.9)	<0.01
Coronary artery disease	196	(26.1)	771	(25.7)	0.01	3,377	(6.5)	3,373	(6.4)	<0.01
Congestive heart failure	122	(16.3)	449	(15.0)	0.04	1,055	(2.0)	1,049	(2.0)	<0.01
Peripheral vascular disease	37	(4.9)	112	(3.7)	0.06	329	(0.6)	326	(0.6)	<0.01
Cerebrovascular disease	92	(12.3)	358	(11.9)	0.01	2,885	(5.5)	2,882	(5.5)	<0.01
Liver cirrhosis	61	(8.1)	226	(7.5)	0.02	2,783	(5.3)	2,783	(5.3)	<0.01
Major cancers	64	(8.5)	238	(7.9)	0.02	7,510	(14.4)	7,510	(14.4)	<0.01
**CCI score**										
0–2	190	(25.3)	757	(25.2)	0.00	43,705	(83.6)	43,710	(83.6)	<0.01
3+	560	(74.7)	2,243	(74.8)	0.03	8,592	(16.4)	8,587	(16.4)	<0.01

A SMD of <0.1 indicates a negligible difference between two groups. CCI, Charlson comorbidity index; HD, hemodialysis; PD, peritoneal dialysis; PSM, propensity score matching; SD, standard deviation; SMD, standardized mean difference.

### Study outcome

The main study outcome was a diagnostic claim for altered mental status in the 30-day period after the index date. We applied a 6-month washout period to ensure that altered mental status constituted a new episode. Altered mental status was diagnosed according to *ICD-9-CM* codes (Supplementary Table 1), which were used in a previous study (23).

### Recommended dosage of valacyclovir in patients with hemodialysis and peritoneal dialysis

Because valacyclovir is excreted through the kidneys, patients with ESRD on dialysis, including HD and PD, might be vulnerable to adverse effects. In patients with normal renal function, the recommended dosage is 1,000 mg of valacyclovir by mouth three times daily for 7 days. One case review indicated that the dosage was reduced to 500 mg/day for patients with HD. However, three patients on HD treated with this reduced dosage developed VAN (4). Furukubo et al. proposed the administration of 500 mg of valacyclovir after every HD session for patients with herpes zoster; this is equivalent to a dosage of 1,500 mg per week (4). Therefore, we evaluated the risk, according to the following dosage categories in patients on HD: overdose (>500 mg/day), recommended dosage A (>1,500 mg/week and ≤500 mg/day), and recommended dosage B (500 mg three times/week). Moreover, we evaluated the risk according to the following dosage categories in patients on PD according to a previous report: overdose (>500 mg/day) and recommended dosage (≤500 mg/day) (10).

### Statistical analysis

Potential confounders at baseline were compared between valacyclovir users and non-users by using the standardized mean difference (SMD) in both cohorts. A SMD of ≤0.1 denotes a negligible imbalance in the potential confounders between the two study groups (21). We employed the Cox proportional hazard model to estimate the risk of the study outcome. Thus, we present the Kaplan–Meier curve and the hazard ratio (HR) for the risk of altered mental status in valacyclovir users compared with non-users. All analyses were performed using SAS/STAT software, Version 9.4 of the SAS System for Unix (SAS Institute Inc., Cary, NC, USA) and STATA 15 software (StataCorp LP, College Station, TX, USA). A two-sided *P*-value of <0.05 was considered significant.

## Results

### Patient characteristics

Among the 2,284,800 patients who had received a diagnosis of herpes zoster between 2002 and 2016, 1,122,754 met the eligibility criteria. The final sample comprised 7,153 patients with ESRD on dialysis and 998,662 patients with normal renal function. Following PSM, we enrolled 750 valacyclovir users and 3,000 valacyclovir non-users in the ESRD cohort and 52,297 valacyclovir users and 52,297 valacyclovir non-users in the normal renal function cohort (Figure 1). Baseline characteristics of the patients are presented in Table 1. In the ESRD cohort, approximately 85% of patients on dialysis were in HD. Compared with patients with the normal renal function cohort, the ESRD cohort had a higher mean age, lower percentage of male patients, and higher comorbidity and CCI scores, except for cancer.

### Valacyclovir-associated neurotoxicity risk

**FIGURE 2:**
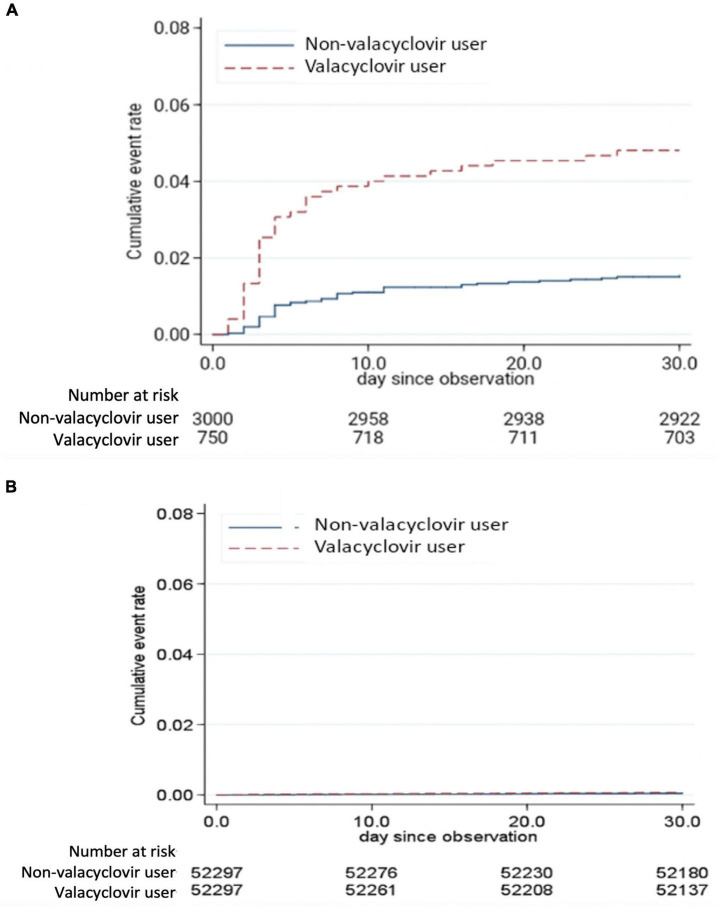
Kaplan–Meier failure curve of altered mental status in the end-stage renal disease cohort **(A)** and normal renal function cohort **(B)** over a 30-day follow-up period.

A Kaplan–Meier failure curve demonstrated that valacyclovir users in the ESRD cohort had a higher risk of developing an altered mental status within 30 days after receiving a diagnosis of herpes zoster than did valacyclovir non-users in the same cohort (Figure 2A). However, no significant difference in the risk of altered mental status was observed between valacyclovir users and non-users in the normal renal function cohort (Figure 2B).

The incidence (per 1,000 person-day) and the risk of altered mental status in patients who did or did not use valacyclovir within the 30-day follow-up period are presented in Table 2. The incidence of altered mental status in users and non-users in the ESRD cohort was 1.68 and 0.52 per 1,000 person-days, respectively (adjusted HR, 3.22; 95% confidence interval [CI]: 2.08–4.99, *P* < 0.001). These results were consistent between the patients with HD and those with PD. The incidence of altered mental status in users and non-users among patients on HD was 1.64 and 0.52 per 1,000 person-days, respectively (adjusted HR, 3.20; 95% CI: 1.98–5.15, *P* < 0.001). The incidence of altered mental status in users and non-users among the patients with PD was 1.85 and 0.53 per 1,000 person-days, respectively (adjusted HR, 3.44; 95% CI: 1.13–10.49, *P* = 0.030). The incidence of altered mental status was extremely low among patients with normal renal function, with no association between valacyclovir use and altered mental status.

**TABLE 2:** Incidence (per 1,000 person-days) and risk of altered mental status among valacyclovir users and non-users over a 30-day period.

Study cohort	Valacyclovir	Number of patients	Number of events	Incidence (95% CI)	Crude HR (95% CI)	*P*	Adjusted* HR (95% CI)	*P*
End-stage renal disease (on dialysis)	Non-user	3,000	46	0.52 (0.39–0.69)	1.00 (Ref.)		1.00 (Ref.)	
	User	750	36	1.68 (1.21–2.32)	3.19 (2.06–4.94)	<0.001	3.22 (2.08–4.99)	<0.001
HD	Non-user	2,558	39	0.52 (0.38–0.71)	1.00 (Ref.)		1.00 (Ref.)	
	User	637	30	1.64 (1.15–2.35)	3.15 (1.95–5.06)	<0.001	3.20 (1.98–5.15)	<0.001
PD	Non-user	442	7	0.53 (0.25–1.12)	1.00 (Ref.)		1.00 (Ref.)	
	User	113	6	1.85 (0.83–4.13)	3.44 (1.16–10.24)	0.026	3.44 (1.13–10.49)	0.030
Normal renal function	Non-user	52,297	22	0.01 (0.01–0.02)	1.00 (Ref.)		1.00 (Ref.)	
	User	52,297	35	0.02 (0.02–0.03)	1.59 (0.93–2.71)	0.088	1.59 (0.93–2.71)	0.088

*The adjusted HR was calculated using the Cox proportional hazard regression model, adjusted for the variables listed in Table 1. CI, confidence interval; HD, hemodialysis; HR, hazard ratio; PD, peritoneal dialysis; Ref., reference.

### Subgroup analysis

The results of the subgroup analysis of patients in the ESRD cohort who were prescribed valacyclovir medication are shown in Table 3. Among the patients on HD, 60.0% were given an overdose of valacyclovir; among those with PD, 54.9% did. Thus, most cases of VAN involved a significantly higher dosage of valacyclovir than recommended. In patients on HD, the incidence of altered mental status was 1.84 and 1.51 per 1,000 person-days in those who were given an overdose (>500 mg/day) and those who were given the recommended dosage A (>1,500 mg/week and ≤500 mg/day), respectively. However, mental status was not altered in patients on HD who were given the recommended dosage B (500 mg three times/week). In patients with PD, the incidence of altered mental status was 3.51 per 1,000 person-days in those who were given an overdose (>500 mg/day). However, mental status was not altered in patients with PD who were given the recommended dosage (≤500 mg/day).

**TABLE 3:** Incidence (per 1,000 person-days) and risk of altered mental status among patients with end-stage renal disease on dialysis receiving different dosages of valacyclovir over a 30-day period.

End-stage renal disease (on dialysis)	Dosage	Number of patients	Number of events	Incidence (95% CI)	Crude HR (95% CI)	*P*	Adjusted HR (95% CI)	*P*
HD	Overdose (>500 mg/day)	380	20	1.84 (1.19–2.86)	1.00 (Ref.)		1.00 (Ref.)	
	Recommended dosage A (>1,500 mg/week and ≤500 mg/day)	230	10	1.51 (0.82–2.81)	0.82 (0.39–1.76)	0.617	0.82 (0.40–1.77)	0.609
	Recommended dosage B (500 mg TIW)	27	0	0				
PD	Overdose (>500 mg/day)	62	6	3.51 (1.58–7.82)				
	Recommended dosage (≤500 mg/day)	51	0	0				

*The adjusted HR was calculated using the Cox proportional hazard regression model, adjusted for the variables listed in Table 1. CI, confidence interval; HD, hemodialysis; HR, hazard ratio; PD, peritoneal dialysis; Ref., reference; TIW, three times per week.

## Discussion

This is the first nationwide population-based study to demonstrate that valacyclovir might increase the risk of altered mental status in patients on HD and PD. More than 50% of patients on HD or PD were given an overdose of valacyclovir. Additionally, most cases of VAN occurred in patients on dialysis being given a significantly higher dosage of valacyclovir than that recommended.

Valacyclovir is an antiviral medication used for treating herpes zoster and herpes simplex infections (24). In multiple case reports, most cases of VAN occurred in patients with impaired renal function (4, 8–11, 25). Orally administered valacyclovir is a prodrug that is converted to acyclovir in the intestine and liver. The bioavailability of valacyclovir per intravenous infusion is 54% vs. approximately 20% per oral dose of acyclovir (26). In patients with normal renal function, valacyclovir is excreted in the urine after 2.5 to 3.3 h, mainly as acyclovir (89%). Neurotoxicity is generally seen with serum acyclovir >3.38 μg/ml although some individuals might be susceptible at lower levels. In a previous study (27) of chronic renal failure patients who receiving 2.5 mg/kg acyclovir as a 1-h infusion, the mean peak acyclovir plasma level at the end of infusion was 8.4 ± 5.4 μg/ml and the level dropped to 0.7 ± 0.3 μg/ml at approximately 48 h. In ESRD patients (28), a large amount of acyclovir is metabolized to 9-carboxymethoxymethylguanine (CMMG), likely through the action of alcohol dehydrogenase and aldehyde dehydrogenase. Hellden et al. (29) showed that serum concentrations of CMMG are consistently increased with VAN in ESRD patients.

Patients who develop VAN might present diverse symptoms, such as confusion, consciousness change, ataxia, or dysarthria. A review article noted that symptoms of disturbance of consciousness (85.0%) and hallucination (45.0%) have the highest incidence in this regard (4). These symptoms of neurotoxicity usually appear within the first 24 to 72 h of treatment. The pathogenesis of VAN is not well established. VAN may be associated with acyclovir primary metabolite, CMMG. In one study, CMMG was detected only in the cerebrospinal fluid (CSF) of patients with VAN (28). CMMG in the CSF might lead to cellular dysfunction in the brain and subsequent neurotoxicity. VAN is a clinical diagnosis and is usually challenging to distinguish from acute encephalitis associated with herpes zoster. If a CSF study confirms encephalitis, patients receive intravenous acyclovir standard treatment.

Reversing VAN requires the discontinuation of the drug involved and emergent HD; in one study, serum valacyclovir levels decreased from 7.4 mcg/mL (average 2–4 mcg/L) to 1.6 mcg/mL after 4 h of HD, thus alleviating VAN. After secondary HD, the serum valacyclovir level further decreased to 0.83 mcg/L without a subsequent rebound (6). Studies have reported that PD could not accelerate recovery from neurotoxicity, indicating that the clearance of valacyclovir through PD was insufficient. However, a recent report observed considerable neurological improvement within a day of intense dialyzate exchanges through PD (9). In this report, when the CAPD dose was increased from four to six exchanges (five exchanges of 2 L of 2.5% dextrose plus one exchange of 2 L of icodextrin), hallucinations were alleviated after 24 h (9). Based on these findings, the intensification of PD therapy could be considered for patients with mild VAN.

For the treatment of herpes zoster in adults, 1,000 mg of valacyclovir three times a day for 7 days is suggested. However, clinicians recommend that this dosage be reduced to 500 mg/day for patients with HD. Patients on valacyclovir might exhibit serum drug accumulation between dialysis sessions. Therefore, Furukubo et al. (4) proposed the administration of 500 mg of valacyclovir after every HD session. In our study, altered mental status was observed in some patients given the recommended dosage A (>1,500 mg/week and ≤500 mg/day). However, mental status was not altered in patients on HD who were given the recommended dosage B 500 mg three times per week. These results suggest that this dosage is suitable for reducing the risk of VAN in patients with HD.

Although PD has a lower valacyclovir clearance rate than HD, PD can enable continuous drug excretion, thereby reducing drug toxicity. Case reports noted that most cases of VAN occurred in patients with renal failure who were given an overdose of valacyclovir (4). In the present study, all VAN cases were reported in patients with PD who were given an overdose of valacyclovir. Therefore, we recommended a dosage of ≤500 mg of valacyclovir per day to lower the risk of VAN in patients on PD.

The risk of valacyclovir overdose–induced VAN in patients with ESRD is preventable through software support systems. For example, an electronic support system can be used to send warnings against overdose to physicians prescribing valacyclovir. Moreover, herpes zoster vaccination could lower the occurrence of herpes zoster in patients with ESRD (30) and also provide protection against VAN.

The current study has several limitations and reimbursement claims data; no laboratory data were collected or examined to classify the patients according to renal function. We employed several strategies to improve the accuracy in cohort grouping; however, we were unable to determine the risk of VAN in patients with mild-to-moderate CKD. Moreover, the accuracy of diagnostic coding might have affected the results. We confirmed the diagnosis of a herpes zoster infection if the patients had at least two diagnostic claims, and we applied a 6-month washout period to ensure that altered mental status was a new episode. Furthermore, because only the data of Taiwanese patients were used, the results might not be generalizable to other populations.

## Conclusion

Our study found that the risk of VAN in patients with HD and PD is significantly higher than that in patients with normal renal function, but this problem is frequently overlooked. Adjusting the valacyclovir dosage according to the recommended value and monitoring adverse effects are crucial for preventing VAN in patients with ESRD who are on dialysis and have herpes zoster.

## Data availability statement

The raw data supporting the conclusions of this article will be made available by the authors, without undue reservation.

## Ethics statement

All personally identifiable information was encrypted before the data were released. Therefore, the Institutional Review Board (IRB) of the Taipei Tzu Chi Hospital (IRB number: 03-W02-091) waived the need for informed consent. Written informed consent for participation was not required for this study in accordance with the national legislation and the institutional requirements.

## Author contributions

Y-CW, S-HJ, C-HL, L-NC, and T-CF designed the study. Y-CW, C-LC, L-YC, L-NC, and T-CF conducted the study. Y-CW, L-NC, and T-CF analyzed the data, performed the statistical analysis, and wrote and edited the manuscript. T-CF takes primary responsibility for the final content of the manuscript. All authors contributed to conceived the study, have read and approved the final manuscript.
