# Supplementary material for: Valacyclovir-associated neurotoxicity among patients on hemodialysis and peritoneal dialysis: A nationwide population-based study
Source: Front Med (Lausanne). 2022 Sep 20;9:997379. doi: 10.3389/fmed.2022.997379 (PMC9530346; doi:10.3389/fmed.2022.997379)
Supplement: Supplementary file 1 [file Table_1.DOCX]

| Supplementary Table 1. Disease diagnostic coding and prescription drug ATC codes | | |
| --- | --- | --- |
| Disease diagnosis | *ICD-9-CM* | *ICD-10-CM* |
| Herpes zoster | 053 | B02 |
| Altered mental status | 292.11, 292.12, 292.2, 292.81, 292.84, 292.89, 292.9, 293.0, 293.1, 298.9, 348.30, 348.39, 349.82, 760.72, 760.77, 780.02, 780.09, 780.1, 780.97 | F05, F10.14, F10.19, F10.24, F10.29, F11.121, F11.14, F11.150, F11.151, F11.159, F11.181, F11.182, F11.188, F11.19, F11.220, F11.221, F11.222, F11.229, F11.24, F11.250, F11.259, F11.281, F11.282, F11.288, F11.29, F11.90, F11.920, F11.921, F11.922, F11.929, F11.94, F11.950, F11.951, F11.959, F11.981, F11.982, F11.988, F11.99, F12.120, F12.121, F12.122, F12.129, F12.150, F12.151, F12.159, F12.180, F12.188, F12.19, F12.220, F12.221, F12.222, F12.229, F12.250, F12.259, F12.280, F12.288, F12.29, F12.920, F12.921, F12.922, F12.929, F12.950, F12.951, F12.959, F12.980, F12.988, F12.99, F13.121, F13.129, F13.14, F13.150, F13.151, F13.159, F13.180, F13.181, F13.182, F13.188, F13.19, F13.220, F13.221, F13.229, F13.24, F13.250, F13.259, F13.280, F13.281, F13.282, F13.288, F13.29, F13.90, F13.920, F13.921, F13.929, F13.94, F13.950, F13.951, F13.959, F13.980, F13.981, F13.982, F13.988, F13.99, F14.121, F14.122, F14.129, F14.14, F14.150, F14.151, F14.159, F14.180, F14.181, F14.182, F14.188, F14.19, F14.220, F14.221, F14.222, F14.229, F14.24, F14.250, F14.259, F14.280, F14.281, F14.282, F14.288, F14.29, F14.90, F14.920, F14.921, F14.922, F14.929, F14.94, F14.950, F14.951, F14.959, F14.980, F14.981, F14.982, F14.988, F14.99, F15.121, F15.122, F15.129, F15.14, F15.150, F15.151, F15.159, F15.180, F15.181, F15.182, F15.188, F15.19, F15.220, F15.221, F15.222, F15.229, F15.24, F15.250, F15.259, F15.280, F15.281, F15.282, F15.288, F15.29, F15.90, F15.920, F15.921, F15.922, F15.929, F15.94, F15.950, F15.951, F15.959, F15.980, F15.981, F15.982, F15.988, F15.99, F16.121, F16.122, F16.129, F16.14, F16.150, F16.151, F16.159, F16.180, F16.183, F16.188, F16.19, F16.220, F16.221, F16.229, F16.24, F16.250, F16.259, F16.280, F16.283, F16.288, F16.29, F16.90, F16.920, F16.921, F16.929, F16.94, F16.950, F16.951, F16.959, F16.980, F16.983, F16.988, F16.99, F17.208, F17.209, F17.218, F17.219, F17.228, F17.229, F17.298, F17.299, F18.120, F18.121, F18.129, F18.14, F18.150, F18.151, F18.159, F18.180, F18.188, F18.19, F18.220, F18.221, F18.229, F18.24, F18.250, F18.259, F18.280, F18.288, F18.29, F18.920, F18.921, F18.929, F18.94, F18.950, F18.951, F18.959, F18.980, F18.988, F18.99, F19.121, F19.122, F19.129, F19.14, F19.150, F19.151, F19.159, F19.180, F19.181, F19.182, F19.188, F19.19, F19.21, F19.220, F19.221, F19.222, F19.229, F19.24, F19.250, F19.259, F19.280, F19.281, F19.282, F19.288, F19.29, F19.90, F19.920, F19.921,  F19.922, F19.929, F19.94, F19.950, F19.951, F19.959, F19.980, F19.981, F19.982, F19.988, F19.99, F28, F29, G92, P04.49, R40.4, R44.0, R44.2, R44.3 |
| Diabetes mellitus | 250 | E08–E11, E13 |
| Coronary artery disease | 414 | I25.1, I25.3–I25.9 |
| Congestive heart failure | 428 | I50 |
| Peripheral vascular disease | 4339, 411, 7854, V434 | I20.0, I24, I65.9, I63.0–I63.2, I96, Z95.820, Z95.828 |
| Cerebrovascular disease | 430–437 | I60–I66,I67.0–I67.2,I67.4–I67.7, I67.81, I67.82, I67.84, I67.89, I67.9, I68, G45.0–G45.2, G45.4–G45.9, G46 |
| Liver cirrhosis | 571 | K70, K73, K74, K75.4, K75.81, K76.0, K76.89, K76.9, R16.2, |
| Major cancers | 140–172.9, 174–195.8, | C0–C6, C70–C76, C7A.0, D03, E31.22, Z51.12 |
| CKD | 582, 583, 585, 586, 588 | N03, N05–N08, N14–N16, N17.1, N17.2, N18.4–N18.6, N18.9, N19, N25, E10.21, E11.21 |
| Medications | ATC codes | |
| Valaciclovir | J05AB11 | |
| Famciclovir | J05AB09 | |
| Acyclovir | J05AB01 | |
| Erythropoietin | B03XA01, B03XA02, B03XA03 | |
| Procedure code |  | |
| HD | 58001C, 58019C, 58020C, 58021C, 58022C, 58023C, 58024C, 58025C, 58029C | |
| PD | 58002C, 58009A, 58009B, 58010A, 58010B, 58011A, 58011AB, 58011B, 58011C, 58012A, 58012B, 58017B, 58017C, 58026C, 58028C | |

Abbreviations: ATC, Anatomical Therapeutic Chemical; HD, hemodialysis; *ICD-9-CM*, *International Classification of Diseases, 9th Revision, Clinical Modification*; *ICD-10-CM*, *International Classification of Diseases, 10th Revision, Clinical Modification*; PD, peritoneal dialysis, CKD, chronic kidney disease
